# Characterization of the CPAP-treated patient population in Catalonia

**DOI:** 10.1371/journal.pone.0185191

**Published:** 2017-09-21

**Authors:** Cecilia Turino, Sandra Bertran, Ricard Gavaldá, Ivan Teixidó, Holger Woehrle, Montserrat Rué, Francesc Solsona, Joan Escarrabill, Cristina Colls, Anna García-Altés, Jordi de Batlle, Manuel Sánchez de-la-Torre, Ferran Barbé

**Affiliations:** 1 Group of Translational Research in Respiratory Medicine, Hospital Universitari Arnau de Vilanova and Santa Maria, IRBLleida, Lleida, Spain; 2 Centro de Investigación Biomédica en Red de Enfermedades Respiratorias (CIBERES), Madrid, Spain; 3 Department of Computer Science, UPC-BarcelonaTech, Barcelona, Spain; 4 Department of Computer Science & INSPIRES, University of Lleida, Lleida, Spain; 5 Sleep and Ventilation Center Blaubeuren, Respiratory Center Ulm, Ulm, Germany; 6 Unit of Bioestatistics and Epidemiology, IRBLleida, Lleida, Spain; 7 Chronic Care Program (Hospital Clínic) & Master Plan for Respiratory Diseases (Ministry of Health, Catalonia) & REDISSEC, Barcelona, Spain; 8 Public Health Department, Government of Catalonia, Barcelona, Spain; Technische Universitat Munchen, GERMANY

## Abstract

There are different phenotypes of obstructive sleep apnoea (OSA), many of which have not been characterised. Identification of these different phenotypes is important in defining prognosis and guiding the therapeutic strategy. The aim of this study was to characterise the entire population of continuous positive airway pressure (CPAP)-treated patients in Catalonia and identify specific patient profiles using cluster analysis.

A total of 72,217 CPAP-treated patients who contacted the Catalan Health System (CatSalut) during the years 2012 and 2013 were included. Six clusters were identified, classified as “Neoplastic patients” (Cluster 1, 10.4%), “Metabolic syndrome patients” (Cluster 2, 27.7%), “Asthmatic patients” (Cluster 3, 5.8%), “Musculoskeletal and joint disorder patients” (Cluster 4, 10.3%), “Patients with few comorbidities” (Cluster 5, 35.6%) and “Oldest and cardiac disease patients” (Cluster 6, 10.2%). Healthcare facility use and mortality were highest in patients from Cluster 1 and 6. Conversely, patients in Clusters 2 and 4 had low morbidity, mortality and healthcare resource use.

Our findings highlight the heterogeneity of CPAP-treated patients, and suggest that OSA is associated with a different prognosis in the clusters identified. These results suggest the need for a comprehensive and individualised approach to CPAP treatment of OSA.

## Introduction

Obstructive sleep apnoea (OSA) is a chronic disorder characterised by recurrent episodes of upper airway collapse during sleep, and affects 5–14% of adults aged 30–70 years [1]. OSA has been linked with increased rates of morbidity and mortality due to its strong association with hypertension, metabolic, cardiovascular and cerebrovascular diseases, and cancer [2,3]. OSA has a negative impact on quality of life, increases the risk of traffic accidents and has an important socioeconomic impact [4,5]. Given these multiple medical and social consequences, OSA could be considered a complex and heterogeneous disorder, deserving of a multidisciplinary approach and personalised treatment. However, there is really only one standard approach to the management of OSA–the application of nocturnal continuous positive airway pressure (CPAP) to splint the upper airways open. CPAP has been shown to improve quality of life and to decrease arterial blood pressure in patients with resistant hypertension [6, 7].

Using distinct types and sources of data, some authors have recently used cluster analysis to identify different phenotypes of OSA patients [8–11]. Cluster analysis allows patients to be grouped according to similar characteristics while maximising differences among different patient groups. Applied to OSA patients, cluster analysis could help to improve knowledge about the condition, confirm known associations with comorbidities, and potentially identify currently unknown associations. In Catalonia, approximately 1% of the general population is currently estimated to be using CPAP. However, there are no clear data on the profiles of OSA patients treated with CPAP, whether there is heterogeneity within this population, and which pathologies or comorbidities might be associated with different patient profiles.

This study characterised the entire CPAP-treated population of Catalonia using cluster analysis in order to define specific profiles based on age, sex, associated comorbidities, mortality and the use of healthcare resources.

## Methods

### Design, setting and study population

This cross-sectional study was conducted in Catalonia (Spain) based on data from the Agency for Health Quality and Assessment of Catalonia (AQuAS). AQuAS is a public entity working under the auspices of Catalonia’s Health Services Department promoting the quality, safety and sustainability of the public Catalan healthcare system. All OSA patients in the Catalan Health Service who were treated with CPAP and had any use of healthcare resources during 2012 and/or 2013 were included in the analysis. Patients receiving CPAP via private health services were excluded because full data were not available. Since all data were anonymised, neither individual patient consent nor ethical approval were required.

### Coding and selection of diseases

The International Classification of Disease version 9 (ICD-9) was used for disease coding at each contact with the Catalan Public Health Service (in primary care, hospital or nursing home).

For this study, we selected a combination of the most frequent diagnoses in our dataset and made a list of the most clinically relevant diagnoses (Table 1) [9]. Several diagnoses were grouped in disease categories in order to facilitate information management. To obtain consistent and clinically relevant patterns of association, and to avoid spurious relationships that could bias the results, we considered only diagnoses with a prevalence of > 1%.

**Table 1:** The most frequent and clinical relevant diagnoses.

Comorbidities	CHARS ICD-9 Diagnosis Code(s)
HIV	042.xx HIV
Malignant neoplasms	140.xx—149.xx Lip, oral cavity and pharynx
	150.xx—159.xx Digestive organs and peritoneum
	160.xx—165.xx Respiratory and intrathoracic organs
	170.xx—176.xx Bone, connective tissue, skin and breast
	179.xx—189.xx Genitourinary organs
	190.xx—199.xx Other locations
	200.xx—208.xx Lymphatic tissues and hematopoietic
Diabetes	250.xx Diabetes mellitus
Dyslipidaemia	272.xx Disorders of lipid metabolism
Obesity	278.xx Overweight, obesity and other types of hyperalimentation
Anaemia	280.xx Anaemia due to iron deficiency
	281.xx Other deficiency anaemia
	282.xx Hereditary haemolytic anaemias
	283.xx Acquired haemolytic anaemias
	284.xx Aplastic anaemia and other medullary insufficiency syndromes
	285.xx Other anaemias and unspecified anaemias
Dementia	290.xx Dementia
Schizophrenic disorders	295.xx Schizophrenic disorders
Mental disorders	296.xx Mood (affective) disorder
	305.xx Drugs without dependence
Anxiety	300.xx Anxiety, dissociative and somatoform disorders
Parkinson's disease	332.xx Parkinson's disease
Hypertension	401.xx Essential hypertension
	402.xx Hypertensive heart disease
	403.xx Chronic hypertensive kidney disease
	404.xx Hypertensive chronic heart and kidney disease
	405.xx Secondary hypertension
Other heart diseases	414.xx Other forms of chronic ischemic heart disease
Dysrhythmia	427.xx Dysrhythmia
Heart failure	428.xx Heart failure
Cerebrovascular diseases	430.xx Subarachnoid haemorrhage
	431.xx Intracerebral haemorrhage
	432.xx Other intracranial haemorrhage and not specified intracranial haemorrhage
	433.xx Stenosis and occlusion of precerebral arteries
	434.xx Occlusion of the brain arteries
	435.xx Transient cerebral ischemia.
	436.xx Poorly-defined acute cerebrovascular disease
	437.xx Other cerebrovascular diseases and other poorly-defined cerebrovascular diseases
	438.xx Late effects of cerebrovascular disease
COPD	490.xx Non specified as acute or chronic bronchitis
	491.xx Chronic bronchitis
	492.xx Emphysema
Asthma	493.xx Asthma
Bronchiectasis	494.xx Bronchiectasis
Pancreatic diseases	577.xx Pancreatic diseases
Chronic renal failure	584.xx Chronic renal failure
Chronic nephropathy	585.xx Chronic nephropathy
Prostatic hyperplasia	600.xx Prostatic hyperplasia
Inflammatory arthritis	714.xx Inflammatory arthritis
Osteoarthrosis	715.xx Osteoarthrosis and related disorders
Other joint disorders	719.xx Other joint disorders and unspecified joint disorders
Back disorders	724.xx Other disorders and unspecified back disorders
Musculoskeletal disorders	726.xx Peripheral tendinitis
Joint disorders	729.xx Other soft tissue disorders
Osteoporosis	733.xx Osteoporosis

List of the diagnoses and group of diagnoses used to perform the Multiple Correspondence Analysis (MCA) and the cluster analysis.

### Statistical analysis

Descriptive statistics of mean (standard deviation) or median [interquartile range (IQR)] were estimated for quantitative variables with a normal or non-normal distribution, respectively, while absolute and relative frequencies were used for qualitative variables. Normal distribution was analysed using the Shapiro-Wilks test.

A Multiple Correspondence Analysis (MCA) was used to reduce dimensionality on the studied diagnoses. The number of dimensions obtained by MCA was identified using the Kaiser's criterion and the Scree test. The individual coordinates obtained with MCA were introduced in a k-means cluster analysis. Cluster analysis has been used to describe homogeneous subgroups (clusters) with similar characteristics (intra-cluster distance minimised), but different from other groups (inter-cluster distance maximised). The final number of clusters was defined on the basis of maximising the ratio between intra-cluster and inter-cluster variance, more specifically Calinsky-Harabasz's criterion. The R statistical software, version 3.3.1, was used for all the analyses.

## Results

### Patient characteristics

**Fig 1:**
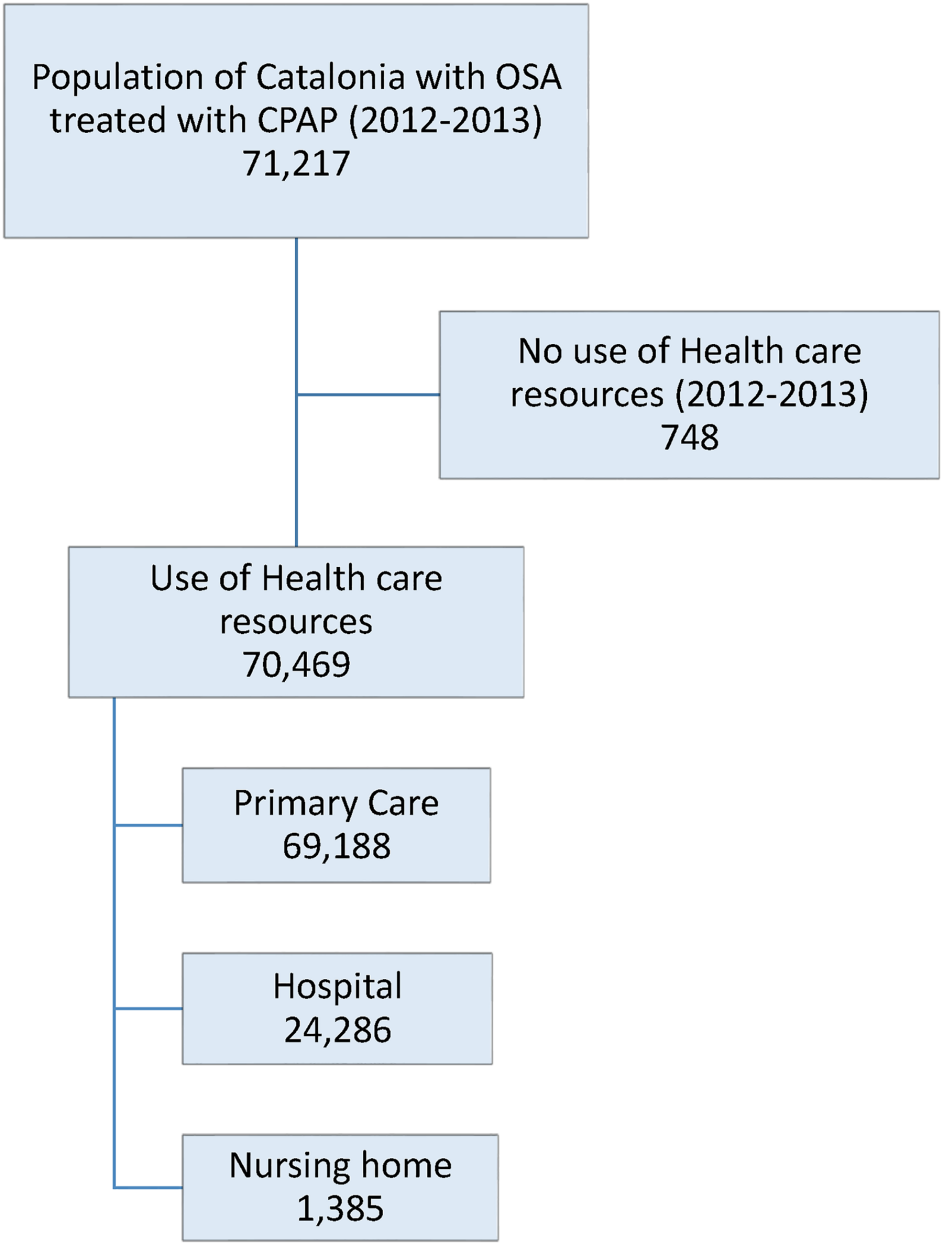
Sampling framework. Abbreviations: OSA, obstructive sleep apnoea; CPAP, continuous positive airway pressure.

Of the 7,478,968 population in Catalonia (2013), 71,217 patients (0.95%) were being treated with CPAP; 70,469 of these used healthcare services (2012–2013) and were included in the analysis (Fig 1). Median age was 64.5 years [IQR57.0; 72.0], 74.9% were men, and 5.29% died during the study period. Median time on CPAP was 34.9 months [IQR 14.8; 58.5]. The most frequent diagnoses were hypertension (61.2%), dyslipidaemia (29.9%), diabetes (29.6%) and obesity (18.3%).

### Cluster analysis

**Fig 2:**
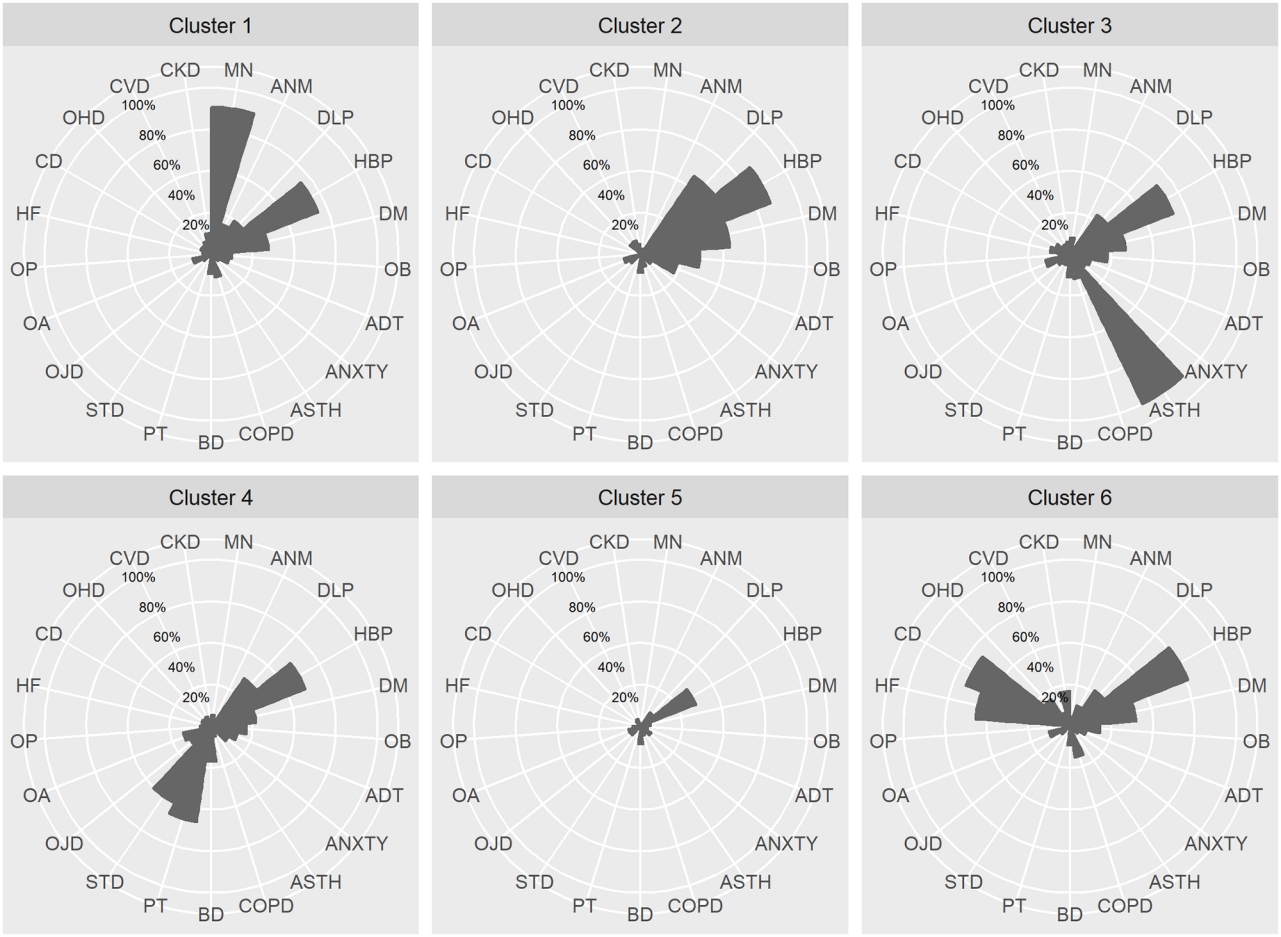
The proportion of each comorbidity in each cluster. Cluster 1: Neoplastic patients. Cluster 2: Metabolic syndrome patients. Cluster 3: Asthmatic patients. Cluster 4: Musculoskeletal and joint disorders patients. Cluster 5: Patients with few comorbidities. Cluster 6: Oldest and cardiac disease patients. Abbreviations: DLP, dyslipidaemia; OB, obesity; BD, back disorders; OA, osteoarthrosis; HF, heart failure; CD, cardiac dysrhythmia; ADT, addiction; ANXTY, anxiety; OHD, other heart disease; OJD, other joint disease; PT, peripheral tendinitis; CKD, chronic kidney disease; ASTH, asthma; STD, soft tissue disease; MN, malignant neoplasm; HBP, hypertension; CVD, cerebrovascular; COPD, chronic obstructive pulmonary disease; ANM, anaemia; DM, diabetes mellitus; OP, osteoporosis.

Six clusters of CPAP-treated OSA patients were identified. Hypertension and diabetes were present in almost all the clusters among the most frequent comorbidities (Fig 2). The main characteristics of each cluster are summarized in Table 2.

**Table 2:** Patient demographic characteristics and annual proportion of health care resource use for the entire cohort and by cluster.

	All clusters	Cluster 1	Cluster 2	Cluster 3	Cluster 4	Cluster 5	Cluster 6	p value
	N = 70,469	N = 7,340	N = 19,535	N = 4,082	N = 7,234	N = 25,088	N = 7,190	
Gender (male)	52805 (74.9%)	5742 (78.2%)	15285 (78.2%)	1917 (47.0%)	4617 (63.8%)	20139 (80.3%)	5105 (71.0%)	<0.001
Age (years)	64.5 [57.0;72.0]	69.5 [62.0;77.0]	64.5 [57.0;69.5]	67.0 [57.0;74.5]	62.0 [57.0;69.5]	62.0 [52.0;67.0]	72.0 [64.5;79.5]	<0.001
CPAP time (months)	34.9 [14.8;58.5]	38.5 [17.9;63.6]	36.8 [15.4;60.8]	32.8 [12.8;55.5]	33.5 [13.7;57.2]	34.2 [14.8;57.4]	32.1 [13.3;56.6]	<0.001
Mortality	3726 (5.29%)	1103 (15.0%)	364 (1.86%)	198 (4.85%)	102 (1.41%)	906 (3.61%)	1053 (14.6%)	<0.001
Nurse home (0 visits)	69084 (98.0%)	6974 (95.0%)	19368 (99.1%)	3949 (96.7%)	7161 (99.0%)	24882 (99.2%)	6750 (93.9%)	<0.001
Nurse home (>0 visits)	1385 (1.97%)	366 (4.99%)	167 (0.85%)	133 (3.26%)	73 (1.01%)	206 (0.82%)	440 (6.12%)	
Hospital (0 visits)	46183 (65.5%)	3335 (45.4%)	14144 (72.4%)	2362 (57.9%)	4768 (65.9%)	18722 (74.6%)	2852 (39.7%)	<0.001
Hospital (1 visit)	13510 (19.2%)	1812 (24.7%)	3407 (17.4%)	841 (20.6%)	1560 (21.6%)	4231 (16.9%)	1659 (23.1%)	
Hospital (>1 visit)	10776 (15.3%)	2193 (29.9%)	1984 (10.2%)	879 (21.5%)	906 (12.5%)	2135 (8.51%)	2679 (37.3%)	
Primary care (0–2.5 visits)	19382 (27.5%)	1410 (19.2%)	3782 (19.4%)	667 (16.3%)	920 (12.7%)	11896 (47.4%)	707 (9.83%)	<0.001
Primary care (2.5–5 visits)	17660 (25.1%)	1721 (23.4%)	5703 (29.2%)	882 (21.6%)	1735 (24.0%)	6679 (26.6%)	940 (13.1%)	
Primary care (>5 visits)	33427 (47.4%)	4209 (57.3%)	10050 (51.4%)	2533 (62.1%)	4579 (63.3%)	6513 (26.0%)	5543 (77.1%)	
Pharmacy (0–1.5 drugs)	24893 (35.3%)	1916 (26.1%)	5251 (26.9%)	916 (22.4%)	2004 (27.7%)	13873 (55.3%)	933 (13.0%)	<0.001
Pharmacy (1.5–3.5 drugs)	22919 (32.5%)	2264 (30.8%)	7879 (40.3%)	1148 (28.1%)	2620 (36.2%)	7336 (29.2%)	1672 (23.3%)	
Pharmacy (>3.5 drugs)	22657 (32.2%)	3160 (43.1%)	6405 (32.8%)	2018 (49.4%)	2610 (36.1%)	3879 (15.5%)	4585 (63.8%)	

Data are presented as median [interquartile range; IQR] and n (%).

Cluster 1: Neoplastic patients. Cluster 2: Metabolic syndrome patients. Cluster 3: Asthmatic patients. Cluster 4: Musculoskeletal and joint disorders patients. Cluster 5: Patients with few-comorbidities. Cluster 6: Oldest and cardiac disease patients.

Cluster 1 (Neoplastic patients) included 7,340 patients (10.4%), a high proportion of whom had malignant neoplasm (88.5%), and the mortality rate was high (15.0%). Cluster 2 (Metabolic syndrome patients) included 19,535 patients (27.7%). High proportions of patients in this group had hypertension (84.1%), dyslipidaemia (57.1%), obesity (35.9%) and diabetes (53.9%); mortality was low (1.9%). Cluster 3 (Asthmatic patients) included 4,082 patients (5.8%). This cluster of patients all had asthma, included a high proportion of women (53.0%) and had a low mortality rate (4.8%). Cluster 4 (Musculoskeletal and joint disorders patients) included 7,234 patients (10.3%), who had peripheral tendinitis (58.2%), joint diseases (15.2%) and muscular disease (51.0%); this group had the lowest mortality rate (1.4%). Cluster 5 (Patients with few comorbidities) grouped the patients with few comorbidities (n = 25,088 patients, 35.6%). Use of healthcare resources by this group was low, as was the mortality rate (3.6%). Cluster 6 (Oldest and cardiac disease patients, n = 7,190, 10.2%) had a median age of 72.0 years [IQR 64.5;79.5], and patients had dysrhythmia (67.5%) and heart failure (57.1%). This group had one of the highest mortality rates (14.6%).

**Fig 3:**
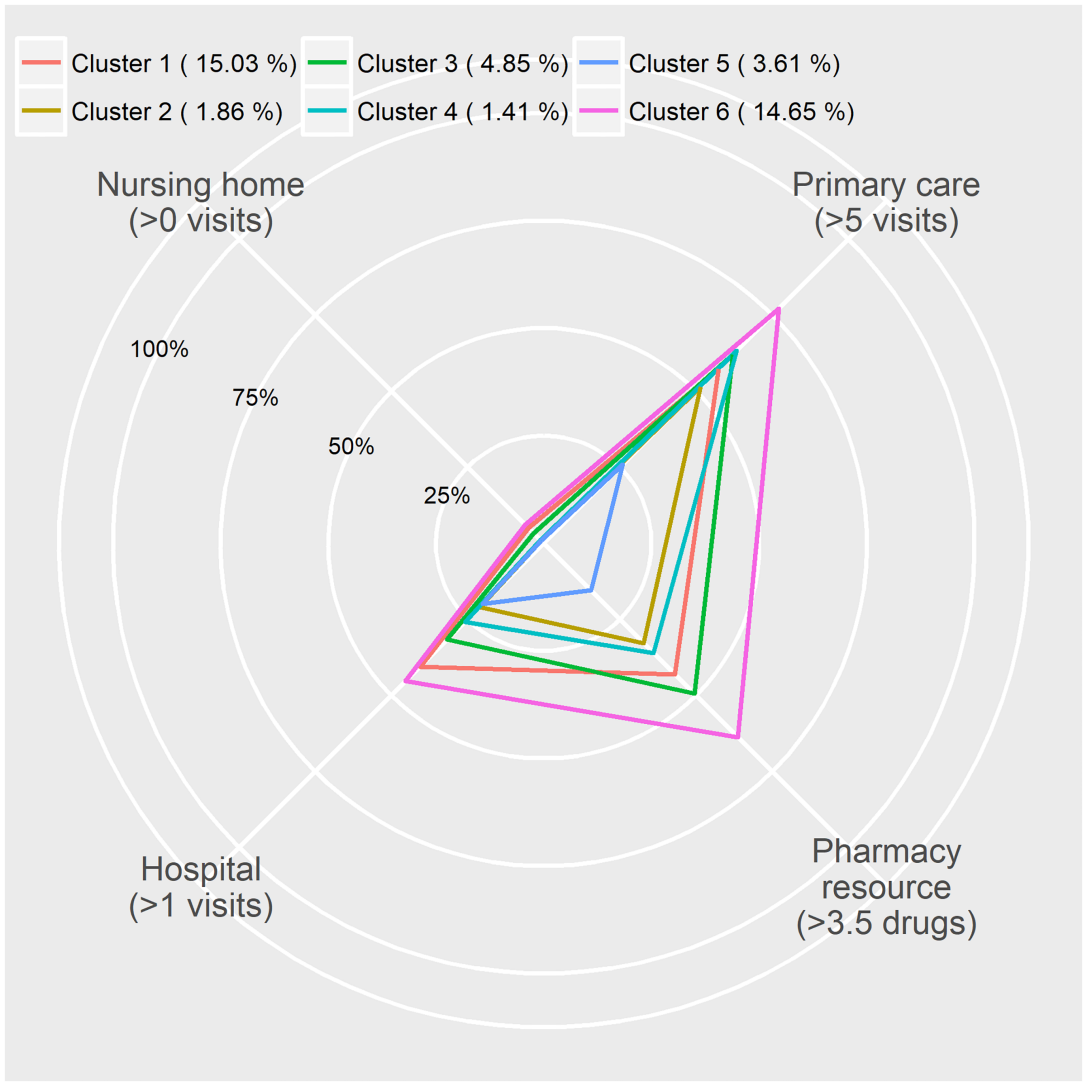
Percentages of high use of health care resources per year in each cluster. Cluster 1: Neoplastic patients. Cluster 2: Metabolic syndrome patients. Cluster 3: Asthmatic patients. Cluster 4: Musculoskeletal and joint disorders patients. Cluster 5: Patients with few comorbidities. Cluster 6: Oldest and cardiac disease patients. (% Mortality).

Mortality rates in both Cluster 1 and 6 were high, but the patient groups differed with respect to the primary comorbidity diagnosis and healthcare resource use. Specifically, patients in Cluster 6 used a wide range of healthcare resources whereas those in Cluster 1 had more hospital and nursing home visits (Fig 3). Mortality rates in Cluster 2 and 4 were also had similar (low), but patients in Cluster 4 used more primary care resources than those in Cluster 2. In addition, mortality rates were similar in Clusters 3 and 5, but healthcare resource use was again different, being very low for Cluster 5 and higher for Cluster 3.

## Discussion

This study is the first cluster analysis involving the entire CPAP-treated population with OSA of Catalonia. Using data from the Catalan Health System, we defined a general profile of OSA patients treated with CPAP, largely characterised by middle-aged men, with a high prevalence of hypertension, dyslipidaemia, diabetes and obesity. At the same time, cluster analysis identified six patient groups that showed different patterns of comorbidities, mortality, and healthcare resource use.

Similar to previous literature, we found that hypertension, diabetes and dyslipidaemia were highly prevalent among OSA patients [12–15]. In a sample of more than 18,000 patients from a prospective national registry, Bailey et al. also confirmed the high burden of comorbidities in OSA patients, identifying six clusters [8]. However, similar to other previous cluster studies, they characterised OSA patients using data from a national registry, clinical practice and sleep registry analysis [8–11], while we exclusively used the coded diseases and discharge data from hospitals, nursing homes and primary care institutions, the number of visits to the emergency room or primary care, and medication use. The observation of a cluster comprised entirely of patients with asthma, mostly women, confirmed previous observations of an OSA-asthma overlap syndrome [16] and suggests a need for more specific studies in this field. Asthma is, in fact, a recognised risk factor for developing OSA [17] and women with OSA are more likely to be diagnosed with asthma [18].

Over and above the characterisation of six clusters, the CPAP-treated population from Catalonia could be divided into two major groups. Almost 20% of the overall population were allocated to clusters 1 and 6, and showed the most advanced age and the highest mortality. The majority of patients (88.5%) in Cluster 1 (Neoplastic patients) had a malignant neoplasm, most frequently of genitourinary or gastrointestinal origin. This is of interest given some current literature reports of a higher prevalence of cancer (particularly pancreatic or renal tumours) in patients with OSA [19]. Cluster 6 (Oldest and cardiac patients) included the oldest individuals (median age 72.0 years), with the highest prevalence of cardiac failure and cardiac arrhythmias. Patients in Clusters 1 and 6 showed the highest mortality and rate of hospitalisation, almost certainly due to the underlying comorbidities rather than as a result of OSA. The presence of cardiovascular diseases has been associated with a worse prognosis in patients using CPAP [20] and CPAP treatment of sleep apnoea has not been shown to improve survival in patients with these comorbidities [21]. Furthermore, in patients over 65 years of age, the presence of OSA seems to have only a slight impact on quality of life, which is determined to a greater extent by the presence of comorbidities [22]. In contrast, more than a half of our population was male, had few comorbidities, and low mortality and healthcare resource use (Clusters 2 and 5). In these groups, OSA appears to be the most important determinant of patient prognosis [23], and patients with these characteristics could be more likely to benefit from CPAP treatment.

Given the different phenotypes identified, the results of our study could have an important impact on Catalan health policies. The Catalan Health System provides free healthcare services to more than 7 million people [24] with annual spending of around €8,000 million [25]. CPAP treatment is provided at no cost to approximately 70,000 people, and cost effectiveness is only achieved after the second year of treatment and exclusively in patients who are compliance with therapy [26]. In Catalonia CPAP therapy is typically prescribed to patients with severe OSA or for more mild disease that is accompanied by daytime hypersomnolence or other symptoms attributable to OSA. Daily CPAP compliance is closely monitored because CPAP treatment is completely free of charge only for patients who used their device for more than 3 hours per night; if this is not the case, CPAP is withdrawn [27]. Even in the absence of specific data about the compliance of our population, it would be reasonable to assume device usage of at least 3 h/night given the treatment criteria and the fact that median duration of CPAP use was 34 months.

Understanding inter-patient differences in clinical presentations of OSA could facilitate more efficient resource management and provision of care. In the light of cluster analysis results, one-third of all CPAP-treated OSA patients in Catalonia (Clusters 1 & 6) are in fact receiving a treatment that probably will not markedly influence their life expectancy or quality of life. Medical resources could be better spent for the remaining population of patients with few comorbidities, for whom a clinical benefit of CPAP treatment would be expected.

This study has several strengths, including the large and comprehensive study population and the statistical method used. In addition, data about patient characteristics, comorbidities and resource use were provided by AQuAS, ensuring high quality information. Furthermore, data were collected from different public health settings (primary care, nursing homes and hospitals) increasing the generalisability of our findings. However, there are some limitations to be considered. Firstly, the absence of clinical information about the study population (e.g. symptoms, quality of life, sleep records, CPAP compliance) limits the ability to fully characterise each cluster. Secondly, use of the ICD-9 classification system reduces the specificity of disease definitions. The ICD-9 sometimes groups similar diseases together, reducing the ability to differentiate between them. However, it does ensure the homogeneity and accuracy of the disease classification. Finally, the absence of a control group also limits our capacity to define clusters and assess whether the features identified are specific to OSA patients.

## Conclusions

This study used cluster analysis based on diagnostic profile to characterise the entire CPAP-treated population of Catalonia for the first time. Six clusters were identified, but the majority of patients could be distributed into two broad groups: one older with high mortality and healthcare resource use, and the other with few comorbidities, low mortality and lower healthcare resource use. Our study highlights the heterogeneity of OSA patients on CPAP treatment, emphasises the importance of identifying the indication and expected benefits of CPAP in specific OSA phenotypes, and offers the opportunity to tailor interventions for specific patient groups.
